# Cytotoxic Activity of Some Medicinal Plants from Hamedan District of Iran 

**Published:** 2014

**Authors:** Sahar Behzad, Atefeh Pirani, Mahmoud Mosaddegh

**Affiliations:** a *School of Pharmacy, Shahid Beheshti University of Medical Sciences, Tehran, Iran*; b *Traditional Medicine and Materia Medica Research Center, Shahid Beheshti University of Medical Sciences, Tehran, Iran. *

**Keywords:** Cytotoxic activity, MTT assay, Iranian medicinal plants, Hamedan

## Abstract

Medicinal plants have been investigated for possible anti-cancer effects. The aim of the present study was to examine the cytotoxic activity of several medicinal plants on different tumor cell lines. 11 selected plant species which have been used in folkloric prescriptions were collected from different sites of Hamedan district of Iran. The methanolic extracts of the plants were prepared and their cytotoxic effects on four human cancer cell lines (A549, human lung adenocarcinoma; MCF7, human breast adenocarcinoma; HepG2, hepatocellular carcinoma and HT-29, human colon carcinoma) and one normal cell line (MDBK, bovine kidney) were examined using the MTT assay. Three of these were exhibited antiproliferative activity against one or more of the cell lines. The extract from *Primula auriculata *demonstrated the highest cytotoxicity with IC50 of 25.79, 35.79 and 43.34 μg.mL−1 against MCF7, HepG2 and HT- 29 cells, respectively. For some of the plants, their traditional use was correlated with the cytotoxic results, whereas for others the results may support the non-cytotoxicity of species used traditionally as natural remedies. The cytotoxic species could be considered as potential of anticancer compounds.

## Introduction

Cancer is one of the main causes of death all over the world. The world health organization (WHO) estimates that 84 million people would die of cancer between 2005 and 2015 (1). Accordingly, much effort has been made to develop various approaches to reduce the threat caused by cancer. Chemotherapy is an important option in modern cancer treatment, and many clinically available anticancer drugs are currently used to treat some types of leukemia, lymphoma and solid tumors (2). 

The introduction of active agents derived from nature into the cancer armamentarium has changed the natural history of many types of human cancer (3, 4).Statistics indicated that a half part of anticancer drugs approved internationally between 1940(s) and 2006 was either natural products or their derivatives (5). 

Iran’s unique meteorological conditions have contributed to the diversity of more than 8000 plant species (6), for this reason many botanists believe the flora of Iran is a green gold (7). Traditional records and ecological diversity indicate that Iranian plants represent an exciting resource for possible lead structures in drug design (6). Local communities in different parts of the country have developed a deep knowledge of various uses of plants during their old history (8). Hamedan district with a long medical tradition and traditional learning of medicinal plants has 315 species of medicinal plants and 59 species are traditionally used (9). It occupies an area of 19,493 km2 and is located in the west of Iran. This district is principally mountainous and plains following the direction of Zagros range. The situated area is located between 34° 47´ and 53° 52´ N ( latitude), 48° 30´ and 52° 56´ E ( longitude) (10).

To find new herbal compounds with anticancer effects, this study focused on selected medicinal plants from Hamedan district of Iran those which have been used in folkloric prescriptions, themselves, or other species from this genus. The selection of plants was based on different literature sources, folklore and traditional medicine. Plants were chosen according to their use against sympatology suggestive of cancer including: abscesses, infected wounds, inflammation, skin disorders, ulcers, perforation.

## Experimental

*Plant material*

*Preparation of extracts*

The aerial part of each plant (100 g) was separated, shade dried and grinded into powder using mortar and pestle at room temperature. Then extracted by maceration with methanol for 72 h. The supernatants were filtered and evaporated under vacuum by means of a rotary evaporator to obtain crude methanolic extracts.

*Cytotoxic assay*

*Cell lines and culture medium*

The following cancer cell lines were used for this study: A549 (human lung adenocarcinoma), MCF7 (human breast adenocarcinoma), MDBK (bovine kidney cells), HepG2 (hepatocellular carcinoma) and HT-29 (human colon carcinoma). Cells were obtained from National Cell Bank of Iran (Pasteur Institute, Tehran, Iran).

MCF7 and HT-29 were cultured in Dulbecco’s modified eagle medium (DMEM; Gibco) with respectively 5% and 10% bovine serum (FBS; Gibco) while other three cell lines were cultured in RPMI 1640 medium (Sigma) with 10% FBS to maintain the desired growth. All cell lines were treated with 1% penicillin-streptomycin (Sigma) in a humidified atmosphere with 5% Co2 at 37 °C throughout the assay.

*MTT assay*

Cell viability was quantified by an MTT colorimetric assay (3-[4, 5-dimethylthiazol-2-yl]-2,5 diphenyl tetrazolium bromide assay) (11). The cells were seeded in 96-well plates at Eleven plant species were collected from Hamedan province of Iran and were identified by botanists at Traditional Medicine and Materia Medica Research Center (TMRC), Shahid Beheshti University of Medical Sciences, Tehran, Iran. A voucher specimen of each species is deposited at TMRC herbarium for future reference (Table 1).

**Table 1:** Selected medicinal plants of Hamedan district, Iran

**Species**	**Family**	**Local name**	**parts Traditionally used**	**Voucher number**
*Alhagi camelorum*	Fabaceae	Taranjabin	Whole plant parts	3258 (TMRC)
*Centaurea aucheri*	Astraceae	Gole gandome	-	3235 (TMRC)
*Centaurea pseudoscabiosa*	Astraceae	Gole gandome	Aerial parts	3233 (TMRC)
*Cerasus microcarpa*	Rosaceae	Albalooye vahshi	Fruits, flowers, core, wood	3259 (TMRC)
*Primula auriculata*	Primulaceae	Tootia	Flower	3224 (TMRC)
*Silene ampulata*	Caryophyllaceae	silene	Aearial parts	3238 (TMRC)
*Silen peduncularis*	Caryophyllaceae	silene	Aearial parts	3229 (TMRC)
*Smyrniopsis aucheri*	Apiaceae	peakal	-	2261 (TMRC)
*Stachys lavandifolia*	Lamiaceae	Toklijeh	Leaves, and flowers	2835 (TMRC)
*Thymus pubescens*	Lamiaceae	Azarbeh	Aerial parts	1593 (TMRC)
*Tripleurospermum disiforme*	Astraceae	Babooneh	Aerial parts	3245 (TMRC)

8.5× 103 for MDBK cells, 7.5 × 103 for MCF7, 15 × 103 for HepG2, 9 × 103 for A549 cells, and 5 × 103 for HT-29 cells and incubated at 37 °C. After 24 h of incubation, when cells reached more than 80% confluence, the medium was removed and the cells were treated with fresh medium containing various concentrations of plant extracts to be tested. Control cells were supplemented with 0.05% DMSO (v/v) vehicle. After 24 h, the supernatants were removed and a fresh medium containing MTT (0.5 mg mL-1) was added to each well at the time of incubation. After 4 h incubation, the supplement was carefully removed, and the remaining formazan crystals were dissolved in DMSO. The plates were shaken for 20 min. The absorbance of each well was measured on an enzyme-linked immunosorbent assay reader (TECAN) at the wavelength of 570 nm.

The dose–response curves of the compounds were fitted by means of the computer program GraphPad Prism 6.0 (GraphPad Software, USA), and IC50 values (the concentration at which the cell proliferation is 50% of the untreated control) were calculated. All *in-vitro *experiments were carried out on two microplates with at least three parallel wells. The antitumor agent 5-FU was used as a positive control in all cell lines. 

## Results

In order to evaluate the cytotoxic effect of 11 plant extracts that are used in Hamedan district of Iran, an antiproliferative assay on four human cancer cell lines (A549, human lung adenocarcinoma; MCF7, human breast adenocarcinoma; HepG2, hepatocellular carcinoma and HT-29, human colon carcinoma) and one normal cell line (MDBK, bovine kidney) was performed. Table 2 presents the list of the investigated plants with traditional uses, chemical constituent and biological activities.

Cytotoxicity activity (IC50) of the eleven plant extracts was shown in Table 3.

Extracts with IC50 > 100 μg.mL-1 in MTT assay were considered inactive.

**Table 2:** Traditional uses, chemical constituents and biological activities of medicinal plants from Hamedan district, Iran

**Species**	**Biological activities**	**Previously isolated compounds**	**Traditional uses**
*Alhagi camelorum*	Antidiarrheal (12), antinociceptive (13), antiulcerogenic (14), gastro protective (15), ureteral stone expulsion (16)	Kampferol, chrysoeriol, isohamnetin, chrysoeriol-7-o-xyloside, kaempferol-3-galacto rhamnoside, iso hamnetin 3-o-ß-D-apio-furanosyl (1-2) ß-D-galactopyranoside (14) alhagitin, alhagidin (17), ferulic acid, isorhamnetin, 5- hydroxymaltol (18), ß-phenethylamine, N-methyl-ß-phenethylamine, N-methyl- tyramine, hordenine, 3:4- dihydroxy-ß-phenethyltrimethylammonium hydroxide, 3-methoxy-4-hydroxy-ß-phenethyltrimethyl ammonium hydroxide, N-methyl mescaline, solsolidine (19)	Gastrointestinal disorders (8, 9), diuretic, wound healing, fever with rash, rheumatic pain (20)
*Centaurea aucheri*	Antioxidant (21)	Caryophyllene oxide, ß-caryophyllene, germacrene-D (22)	-
*Centaurea pseudoscabiosa*	Antibacterial (23)	chrysin, baicalein 6-methyl ether, protocatechuic acid, 5-caffeoyl quinic acid, hispidulin, chrysin 7-O-glucuronide, chrysin 7-O-glucuronide methyl ester, chrysin 6-C-glucoside, chrysin 8-C-glucoside, luteolin 7-glucoside, pinocembrin 7-O-α-arabinopyranosiyl-(1→2)-β-glucopyranoside, chrysin 7-O-β galactopyranuronoside, baicalein 6-methylether-7-O-β-galactopyranuronoside, scopoletin (24) germacrene_D, β-caryophyllene, biocyclogermacrene, β-sesquiphellandrene (25)	Skin ailments (23)
*Cerasus microcarpa*	-	Alkaloide, tannin (26)	Carminative, cure for pains of digestive system(8)
*Primula auriculata*	Antibacterial, antioxidant (27)	Saponin, flavonoid (27)	Flu and sneezing (28), eye diseases, anti-infection, cataract, trachoma (29)
*Silene ampulata*	-	-	Insect repellent(8)
*Silen peduncularis*	-	-	Insect repellent(8)
*Smyrniopsis aucheri*	antibacterial, antifungal (30)	α-bisabolol (31), pcymene, caryophyllene oxide, spathulenol (32), smyrindiol, smyrinol, smyrindioloside (33), smyriniodidin (34),α-pinene, β-pinene, nachsmyrin (30)	-
*Stachys lavandifolia*	Gastroprotective (35), wound healing (36) , analgesic and antiinflammation (37), anxiolytic (38), antimicrobial (39), abortive effect (40)	α-thujene, α-pinene, β-myrcene, β-phellandrene, germacrene-D, cadinene, 1,4-methano-1 H-indene, γ-elemene benzaldehyde (41), apigenin, luteolin (42), lavandulifolioside A, lavandulifolioside B, verbascoside, leucosceptoside A, 5-O-β-allopyranosyloxy-aucubin (43)	Skin infection, menorrhagia, antibacterial (44, 45),gastrointestinal and respiratory disorder (46-48), wound healing, cardiac disorders, fever and malaria (49)
*Thymus pubescens*	Antioxidant (50), antibacterial (51) analgesic and anti-inflammatory (52)	carvacrol, thymol, γ-terpinene, ρ-cymen (53)	gastrointestinal disorder (54), herpes, lung infection and skin problem (55)
*Tripleurospermum disiforme*	Anti-ulcer (56), antibacterial (57),anti-inflammatory, analgesic (58), antioxidant (59), antifungal (60)	Flavonoid (61), β-farensene, β-sesquiphellandrene, ρ-methoxy-β-cyclopropylstyrene,heptadecane, ρ-methoxy-humulene oxide and benzene acetaldehyde (57)	Antispasmodic, anti-inflammatory, Acne and Itching (61)

**Table 3:** *In-vitro *cytotoxicity of methanol extracts of selected medicinal plants

**Species**	**Yields (%)**	**IC50 value (μg.mL** ^-1^ **)**					
		***A549***	**MCF7**	**HepG2**		**HT-29**	**MDBK**
*Alhagi camelorum*	11.3	>100	>100		>100	>100	>100
*Centaurea aucheri*	9.6	>100	>100		53.31	>100	>100
*Centaurea pseudoscabiosa *subsp *pseudoscabiosa*	11.77	54.82	>100		>100	98.15	>100
*Cerasus microcarpa*	10.86	>100	>100		>100	>100	>100
*Primula auriculata*	12	>100	25.79		35.79	43.34	>100
*Silene ampulata*	7.24	>100	>100		>100	>100	>100
*Silen peduncularis*	9.7	>100	>100		>100	>100	>100
*Smyrniopsis aucheri*	19.03	>100	>100		>100	>100	>100
*Stachys lavandifolia*	15.53	>100	>100		>100	>100	>100
*Thymus pubescens*	8.6	>100	>100		>100	>100	>100
*Tripleurospermum disiforme*	11.25	>100	>100		>100	>100	>100

## Discussion

Our study describes investigations into the anticancer potential of 11 so far not studied Iranian medicinal plants by screening for cytotoxic activity against normal bovine kidney and four human cancer cell lines. All plant extracts showed no toxicity against normal bovine kidney, but methanolic extract of *Centaurea aucheri*, *Centaurea pseudoscabiosa *subsp *pseudoscabiosa *and *Primula auriculata *was expected to show more cytotoxic activity against the tumor cells, whereas others were not cytotoxic against any of the cell lines tested.

The large genus *Centaurea *comprises about 500 species, which are predominantly distributed around the Mediterranean area and in west Asia (22).In Iran this genus has 74 annual to perennial herbaceous species that are widespread around the country (62). Several species of the genus *Centaurea *are well known for their traditional medicinal uses for the treatment of a number of ailments including bacterial infections, cancers, diabetes, diarrhea, fever, hypertension, malaria, rheumatism and tumors (63). Many reports showing the existence of various cytotoxic compounds in different *Centaurea *species, including alkaloids, flavonoids, lignans, sesquiterpenes and simple phenolics (64-66). 

Methanol extract from *Centaurea aucheri *displayed selective cancer cell line cytotoxicity with IC50 values of 53.31 μg.mL-1against hepatocellular carcinoma. This plant has not previously been used as anticancer treatment in traditional Iranian medicine and was selected because of cytotoxic effect of this genus.

Some flavonoids and their glycoside isolated from *Centaurea pseudoscabiosa *subsp *pseudoscabiosa *such as chrysin, hispidulin and luteolin (24).these compounds have been shown Significant cytotoxic and apoptotic effects of on various cancer cell lines (MCF7, Hela, HL-60 and KYSE-510)(67-69) and it may be involved in cytotoxic effect of methanolic extract of this species in this study. Interestingly, the plant has been used traditionally in skin ailments (23), however, no anticancer or cytotoxic activities have been reported to date.

Methanolic extract of *Primula auriculata *showed significant cytotoxic activity against breast, liver and colon cancer cell lines with IC50 values ranging from 25.79 to 43.34 μg.mL-1. *Primula auriculata *from Primulaceae family is one of the most important local medicinal plants in Hamedan district (locally named Tootia). White powders that were produced by plant inflorescences named Tootia have been used traditionally for eye infectious diseases (29). In turkey dried herb was sniffed into nose for sneezing to ease respiration in flu (28). The aerial part of this genus are rich in flavonoid (70) and they may be related to cytotoxic effect of *Primula auriculata *methanolic extract.

8 of 11 selected plants showed no cytotoxic activity against normal and cancer cell lines that has a great significance for their traditional use in the treatment of various disorders other than cancer.

This is the first time that methanolic extracts from the 11 listed Iranian plants (*Alhagi camelorum, Centaurea aucheri, Centaurea pseudoscabiosa *subsp *pseudoscabiosa, Cerasus microcarpa, Primula auriculata, Silene ampulata, Silen peduncularis, Smyrniopsis aucheri, Stachys lavandifolia, Thymus pubescens *and *Tripleurospermum disiforme*) have been screened against human lung, liver, colon and breast cancer cell lines and one normal cell line. This study provides an important basis for further investigation into the isolation, characterization and mechanism of cytotoxic compounds from some of the screened Iranian medicinal plants. Thus, these plants could be used as a source for new lead structures in drug design to combat cancer.

## References

[1] Danhier F, Feron O, Preat V (2010). To exploit the tumor microenvironment: passive and active tumor targeting of nanocarriers for anti-cancer drug delivery. J. Control. Release.

[2] Pan L, Chai H, Kinghorn AD (2010). The continuing search for antitumor agents from higher plants. Phytochem. Lett.

[3] Rocha AB, Lopez RM, Schwartsmann G (2001). Natural products in anticancer therapy. Curr. Opin. Pharmacol.

[4] Shiezadeh F, Mousavi S, Amiri M, Iranshahi M, Tayarani-Najaran Z, Karimi G (2013). Cytotoxic and apoptotic potential of Rheum turkestanicum janisch root extract on human cancer and normal cells. Iran. J. Pharm. Res.

[5] Newman DJ, Cragg GM (2007). Natural products as sources of new drugs over the last 25 years. J. Nat. Prod.

[6] Ghannadi A, Zolfaghari B, Shamashian S (2011). Necessity, importance and applications of traditional medicine in different ethnic. J. Tradition. Med. Islam. Iran.

[7] Mosaddegh M, Naghibi F (2002). Iranian Traditional Medicine, Past and Present in Traditional Medicine and Materia Medica.

[8] Mosaddegh M, Naghibi F, Moazzeni H, Pirani A, Esmaeili S (2012). Ethnobotanical survey of herbal remedies traditionally used in Kohghiluyeh va Boyer Ahmad province of Iran. J. Ethnopharmacol.

[9] Kalvandi R, Safikhani K, Najafi, Babakhanlo P (2007). Identification of medicinal plants of Hamedan province. Iran. J. Med. Aromatic Plants.

[10] Fereidounfar F, Shirzadian S, Ranjbar M, Ghahremaninejad F (2011). A survey to the moss flora of Alvand mountains in Hamedan province, W Iran. Iran. J. Botany.

[11] Mosmann T (1983). A Rapid Colorimetric Assay for Cellular Growth and Survival: Application to Proliferation and Cytotoxic Assay. J. Immunol. Methods.

[12] Atta AH, Mounier SM (2004). Antidiarrhoeal activity of some Egyptian medicinal plant extracts. J. Ethnopharmacol.

[13] Atta AH, Abo El-sooud K (2004). The antinociceptive effect of some Egyptian medicinal plant extracts. J. Ethnopharmacol.

[14] Awaad Amani AS, Maitland DJ, Soliman GA (2006). Antiulcerogenic Activity of Alhagi maurorum. Pharm. Biol.

[15] Gharibnaseri MK, Mard SA (2007). Gastroprotective effect of Alhagi camelorum on experimental gastric ulcer in rats. Physiol. Pharmacol.

[16] Cyrus A, Goodarzi D, Jahangiri V (2010). The effect of Alhagi Pseudalhagi distillate on ureteral stone expulsion. Arak Med. Uni. J.

[17] Singh VP, Yadav B, Pandey VB (1999). Flavanone glycosides from Alhagi pseudalhagi. Phytochem.

[18] Sultan A, Moohammadnor M, Eshbakova K (2011). Chemical constituents of Alhagi pseudalhagi. Chem. Nat. Compd.

[19] Ghosal S, Srivastava RS, Bhattacharya SK, Debnath PK (1974). The Active Principles of Alhagi pseudoalhagi: β-Phenethylamine and Tetrahydroisoquinoline Bases. Planta. Med.

[20] Shafiezadeh F (2002). Medicinal plants of Lorestan.

[21] Hajimehdipoor H, Bandidarian A, Hamzeloo Moghadam M, Mosaddegh M (2012). Antioxidant property of two Star Thistles (Centaurea) from Iran. Res. Pharm. Sci.

[22] Asadipour A, Mehrabani M, Lari Najafi M (2005). Volatile oil composition of Centaurea aucheri (DC). Wagenitz. Daru.

[23] Uysal I, Celik S, Oldacay M (2005). Antibacterial Activity of Centaurea Species Having Ethnobotanical Features. Pak. J. Pharm. Sci.

[24] Flamini G, Pardini M, Morelli L, Ertugrul K, Dural H, Bagci Y, Kargioglu M (2002). Flavonoid glycosides from Centaurea pseudoscabiosa subsp. pseudoscabiosa from Turkey. Phytochem.

[25] Flaminia G, Ertugrul K, Cioni PL, Morelli I, Dural H, Bagci Y (2002). Volatile constituents of two endemic Centaurea species from Turkey: C. pseudoscabiosa subsp. pseudoscabiosa and C. hadimensis. Biochem. Syst. Ecol.

[26] Alikayani s, Masood A, Achakzai AKK, Anbreen S (2007). Distribution of Secondary Metabolites In Plants of Quetta-Balochistan. Pak J Bot.

[27] Jaberian H, Piri K, Nazari J (2013). Phytochemical composition and in vitro antimicrobial and antioxidant activities of some medicinal plants. Food Chem.

[28] Sezik E, Yesilada E, Honda G, Takaishi Y, Takeda Y, Tanaka T (2001). Traditional medicine in Turkey X. Folk medicine in Central Anatolia. J. Ethnopharmacol.

[29] Najafi G, Kalvandi R, Safikhani K (2004). Presentation of native knowledge and new finding about medicinal plant of Primula auriculata.

[30] Faridi P, Ghasemi Y, Gholami A, Mehregan I, Mohagheghzadeh A (2008). Antimicrobial Essential Oil from Smyrniopsis Aucheri. Chem. Nat. Comp.

[31] Kamatou GPP, Viljoen AM (2010). A review of the application and pharmacological properties of α-bisabolol and α-bisabolol-rich oils. J. Am. Oil Chem. Soc.

[32] Esmaeili A, Amiri H, Rustaiyan A, Masoudi S, Tabatabaei-Anaraki M (2010). The essential oils of two umbelliferae, Zosimia absinthifolia (Vent.) link. and smyrniopsisaucheri boiss. Grwoing wild in Iran. J. Essent. Oil Bear. Pl.

[33] Dzhafarov ZR, Kuliev ZA, Vdovin AD, Kuliev AA, Malikov VM, Ismailov NM (1192). Coumarins of Smyrniopsis aucheri. Chem. Nat. Comp.

[34] Savina AA, Perel'son ME, Nikonov GK (1973). The structure of smyrnioridin. Chem. Nat. Comp.

[35] Nabavizadeh F, Alizadeh AM, Adeli S, Golestan M, Moloudian H, Kamalinejad M (2011). Gastroprotective effects of Stachys Lavandulifolia extract on experimental gastric ulcer. Afr. J. Pharm. Pharacol.

[36] Ghasemi Pirbalouti A, Koohpyeh A (2011). Wound healing activity of extracts of Malva sylvestris and Stachys lavandulifolia. Int. J. Biol.

[37] Hajhashemi V, Ghannadi A, Sedighifar S (2007). Analgesic and Anti-inflammatory Properties of the Hydroalcoholic, Polyphenolicand Boiled Extracts of Stachys lavandulifolia. Res. Pharm. Sci.

[38] Rabbani M, Sajjadi SE, Zarei HR (2003). Anxiolytic effects of Stachys lavandulifolia Vahl on the elevated plus-maze model of anxiety in mice. J. Ethnopharmacol.

[39] Saeedi M, Morteza-Semnani K, Mahdavi MR, Rahimi F (2008). Antimicrobial studies on extracts of four species of stachys. Indian J. Pharm. Sci.

[40] Jafarzadeh L, Rafieian-Kopaei M, Samani RA (1012). The effect of hydroalcoholic extract of Stachys lavandulifolia vahl on pregnant mice. EXCLI J.

[41] Pirbalouti AG, Malekpoor F, Mohammadi M, Yousefi M (2012). Composition of the essential oil of Stachys lavandulifolia from central Zagros Mountains. Acta. Horticulturae.

[42] Safaei A (2004). Identification and Quantitative Determination of Luteolin and Apigenin in Aerial Parts of Stachys lavandulifolia by HPLC.

[43] Delazar A, Delnavazi MR, Nahar L, Moghadam SB, Mojarab M, Gupta A, Williams AS, Mukhlesur Rahman M, Sarker SD (2011). Lavandulifolioside B: a new phenylethanoid glycoside from the aerial parts of Stachys lavandulifolia Vahl. Nat. Prod. Res.

[44] Pirbalouti AG (2009). Medicinal plants used in Chaharmahal and Bakhtyari districts of Iran. Herba Polonica.

[45] Zargari A (1989-1992). Medicinal Plants.

[46] Işcan G, Demirci B, Demirci F, Gog̈er F, Kirimer N, Köse YB, Başer KHC (2012). Antimicrobial and antioxidant activities of Stachys lavandulifolia subsp. lavandulifolia essential oil and its infusion. Nat. Prod. Commun.

[47] Amin G (1991). Popular medicinal plants of Iran.

[48] Ahvazi M, Mozaffarian V, Nejadsatari T, Mojab F, Charkhchian MM, Khalighi-Sigaroodi F, Ajani Y (2009). Medicinal application of native plants (Lamiaceae and Rosaceae family) in Alamut region in Gazvin province. J. Med. Plants.

[49] Mozaffarian V (2012). Identification of medicinal and aromatic Plants of Iran.

[50] Poumohamad F, Enteshari S, Sariri R (2011). Total phenolic content and antioxidant activity of the methanolic extracts of three Thymus cultivars grown in Iran. Pharmacologyonline.

[51] Rasooli I, Mirmostafa SA (2002). Antibacterial properties of Thymus pubescens and Thymus serpyllum essential oils. Fitoterapia.

[52] Mahmoudi M, Morteza-Semnani Kand Mojra E (2008). Anti-Inflammatory and Antinociceptive Activity of Thymus pubescens Extract. Fitoterapia.

[53] Sefidkon F, Askari F, Ghorbani M (2002). Essential oil composition of Thymus pubescens Boiss. et Kotschy ex Celak from Iran. J. Essent. Oil Res.

[54] Nazemiyeh H, Lotfipoor F, Delazar A, Razavi SM, Asnaashari S, Kasebi N, Talebpour A-H, Nahar L, Sarker SD (2011). Chemical composition, and antibacterial and free-radical scavenging activities of the essential oils of a citronellol producing new chemotype of Thymus pubescens Boiss. & Kotschy ex Celak. Rec. Nat. Pro.

[55] Rahimi- Golsefidi R (2010). Medicinal plants of Zagros Bakhtiari.

[56] Minaiyan M, Ghassemi dehkordi N, Mohammadzadeh M (2006). Anti-ulcer effect of Tripleurospermum disforme (M.A.Mey)Shylts Bip on pylorus ligated (Shay) rats. Res. Pharm. Sci.

[57] Chehregani A, Mohsenzadeh F, Mirazi N, Hajisadeghian S, Baghali Z (2010). Chemical composition and antibacterial activity of essential oils of Tripleurospermum disciforme in three developmental stages. Pharm. Biol.

[58] Parvini S, Hosseini MJ, Bakhtiarian A (2007). The study of analgesic effects and acute toxicity of Tripleurospermum disciforme in rats by formalin test. Toxicol. Mech. Methods.

[59] Souri E, Sarkhail P, Kaymanesh P, Amini M, Farsam H (2005). Antioxidant activity of extract and a new isolated dioxaspiran derivative of Tripleurospermum disciforme. Pharm. Biol.

[60] Amin G, Dehmoobed Sharif-Abadi A, Salehi Soormaghi MH, Yasa N, Aynechi Y, Emami M, Shidfar M, Amin M, Moghadami M (2002). Screening of Iranian plants for anti-fungal activity: part1. Daru.

[61] Ghasemi Dehkordi N, Amin G, Rahiminezhad MR, Salehi MH, Jafarpisheh A (2003). Morphological and phytochemical study of Tripleurospermum disciforme (C.A.MEY) Schultz Bip. Pajouhesh-va-Sazandegi.

[62] Mozaffarian V (1996). A dictionary of Iranian plant names.

[63] Arif RK, Reyhan; Ergun, Fatma (2004). The Biological Activity of Centaurea L. Species. GU J. Sci.

[64] Chicca AT, Marianna; Adinolfi, Barbara; Ertugrul, Kuddisi ; Flamini, Guido;Nieri, Paol (2011). Anti-proliferative activity of aguerin B and a new rare nor-guaianolide lactone from arial part of Centaurea deflexa. Eur. J. Med. Chem.

[65] Forgo PZ, István; Molnár, Judit; Vasas, Andrea; Dombi, György; Hohmann, Judit (2012). Bioactivity-guided isolation of antiproliferative compounds from centaurea jacea L. Fitoterapia.

[66] Kolli EHL, Francisco; Benayache, Fadila; Estévez, Sara; Quintana, José (2012). Cytotoxic Sesquiterpene Lactones and other Constituents of Centaurea omphalotricha. J. Braz. Chem. Soc.

[67] Khoo BYC, Siang Ling, Balaram, Prabha (2010). Apoptotic Effects of Chrysin in Human Cancer Cell Lines. Int. J. Mol. Sci.

[68] He LW, YLin L, Wang J, Wu Y, Chen Y, Yi Z, Liu M, Pang X (2011). Hispidulin, a small flavonoid molecule, suppresses the angiogenesis and growth of human pancreatic cancer by targeting vascular endothelial growth factor receptor 2-mediated PI3K/Akt/mTOR signaling pathway. Cancer Sci.

[69] Csupor-Loffler B, Hajdu Z, Zupko I, Rethy B, Falkay G, Forgo P, Hohmann J (2009). Antiproliferative effect of flavonoids and sesquiterpenoids from Achillea millefolium s.l. on cultured human tumour cell lines. Phytother. Res.

[70] Fico G, Rodondi G, Flamini G, Passarella D, Tome F (2007). Comparative phytochemical and morphological analyses of three Italian Primula species. Phytochem.

